# Anxiety, depression, and medication adherence in Chinese patients with myocardial infarction in the absence of obstructive coronary artery disease

**DOI:** 10.1002/clc.23495

**Published:** 2020-10-26

**Authors:** Menglu Liu, Chao Deng, Ping Yuan, Jianyong Ma, Peng Yu, Jie Chen, Yujie Zhao, Xiao Liu

**Affiliations:** ^1^ Department of Cardiology The Seventh People's Hospital Zhengzhou Henan China; ^2^ Cardiology Department Affiliated Hospital of Jiangxi University of Traditional Chinese Medicine Jiangxi China; ^3^ Cardiology Department Sun Yat‐sen Memorial Hospital of Sun Yat‐sen University Guangzhou Guangzhou China; ^4^ Department of Pharmacology Systems Physiology University of Cincinnati College of Medicine Cincinnati Ohio USA; ^5^ Endocrine Department The Second Affiliated Hospital of Nanchang University Jiangxi China; ^6^ Cardiology Department The Third Affiliated Hospital of Nanchang University Jiangxi China

To the Editor,

We read the article by He et al.^1^ about the effect of anxiety status on the patients' adverse outcomes with myocardial infarction in the absence of obstructive coronary artery disease (MINOCA) with great interest. They demonstrated that anxiety status was significantly and independently associated with an increased risk of all‐cause mortality and major adverse cardiovascular events in patients with MINOCA. However, we have several concerns regarding their results. Firstly, as described by the authors in the introduction, anxiety, and depression were the common psychological syndromes in patients with cardiovascular diseases (CVDs).^2^ Numbers of studies have demonstrated that anxiety and depression were associated with an increased adverse outcome in patients with CVDs,^2^ as well as in patients with MINOCA.^3^ Moreover, anxiety and depression are frequent coexisting.^4^ In China, an estimated 49% of patients with anxiety had a comorbid depressive disorder according to a cohort that included 85 465 patients.^5^ However, in He et al.'s^1^ report, depression status was not described in the baseline characteristics and adjusted in the multivariate cox regression model. This raised the concern of whether the increased risk of mortality independently attributed to the anxiety, especially under the condition of the association between anxiety and adverse outcomes was not strong as described in their conclusions. The current data are insufficient to support their findings. Secondly, the anxiety group might has lower adherence to medication in He et al.'s cohort. Numbers of studies have reported that anxiety and depression were significantly associated with poor medication adherence.^6^ And poor medication adherence significantly increased the risk of adverse outcomes in patients with CVDs. For example, according to a recently study, almost 31% of patients with myocardial infarction have not persistent with their prescribed medications by six months.^7^ A meta‐analysis included 1 978 919 patients showed that only 60% of patients were adherence to their cardiovascular medications. Besides, compared with good adherence, the risk of cardiovascular events or mortality increased by 20% or 38%, respectively,^8^ in those with poor adherence. Therefore, the effect of medication adherence should be further assessed and adjusted in their results, instead of merely adjusting for the use of medications at discharge. Overall, these issues above might be further discussed to increase the strength and robustness of their conclusion.

## CONFLICT OF INTEREST

The authors declare no potential conflict of interests.
